# Potential Anticancer Activity of Crude Ethanol, Ethyl Acetate, and Water Extracts of *Ephedra foeminea* on Human Osteosarcoma U2OS Cell Viability and Migration

**DOI:** 10.1155/2020/3837693

**Published:** 2020-07-09

**Authors:** Eric Zadok Mpingirika, Ahmed El Hosseiny, Sheri Magdy Saleeb Bakheit, Rami Arafeh, Asma Amleh

**Affiliations:** ^1^Biotechnology Program, American University in Cairo, New Cairo 11835, Egypt; ^2^Department of Biology, American University in Cairo, New Cairo 11835, Egypt; ^3^Palestine-Korea Biotechnology Center, Palestine Polytechnic University, Hebron, State of Palestine

## Abstract

Medicinal plants are potential sources for a wide range of complex compounds with probable anticancer activity. *Ephedra foeminea* Forssk. (*E. foeminea*), a medicinal plant found in the Eastern Mediterranean, has recently been gaining popularity as a cancer remedy; there is, however, a paucity of empirical evidence supporting this claim. In this study, the effect of *E. foemine*a ethyl acetate, ethanol, and water crude extracts on viability, migratory ability, and the steady-state mRNA levels of genes involved in these processes was, respectively, examined using MTT assay, wound healing assay, and reverse transcriptase PCR (RT-PCR). The study concludes that all extracts significantly reduce human osteosarcoma U2OS percentage viability in a dose- and time-dependent manner, with varying potencies. The least half-maximal inhibitory concentration (IC_50_) was observed in the water extract after 48 h incubation (30.761 ± 1.4 *μ*g/mL) followed by the ethyl acetate extract after 72 h incubation (80.35 ± 1.233 *μ*g/mL) and finally the ethanol extract after 48 h incubation (97.499 ± 1.188 *μ*g/mL). Ethanol extract significantly reduced U2OS percentage wound closure. On the other hand, both ethanol and water extracts considerably reduced the steady-state mRNA expression of *beta-catenin*, promoting both cell proliferation and migration in osteosarcoma by regulating target genes. Additionally, *E. foeminea* showed no hemolytic activity. These effects suggest that *E. foeminea* decreases U2OS cell viability and migratory ability by modulating the expression of critical genes involved in regulating these processes and is likely cytocompatible with human erythrocytes.

## 1. Introduction

Accounting for approximately a sixth of all deaths worldwide, the World Health Organization ranks cancer as the second leading cause of death in the world. With 14 million new cases recorded in 2012, a 70% increase in such incidences is expected within the next twenty years [1]. Despite the progress made in developing cancer treatment procedures, contemporary cancer remedies including surgery and chemo- and radiotherapy remain both expensive and ineffective; they pose several adverse side effects to patients, along with the development of drug resistance [2].

Osteosarcoma is the most common primary malignant bone tumor prevalent in both children and adolescents. It accounts for approximately 5% of all pediatric malignancies and has a global incidence of about 4.5 cases per 1 million individuals annually. Osteosarcoma treatment is administered through a combination of both surgery and chemotherapy, with patient survival rates of about 70% [3]. The U2OS human osteosarcoma cell line utilized in this study has been reported to be moderately differentiated, with highly altered chromosomes that exist in the hypertriploid range [4].

Several markers involved in cell proliferation, migration, and apoptosis have been implicated in osteosarcoma. In this study, the expression of beta-catenin, a key regulator of proliferation in osteogenic cells, was assessed, and its steady-state mRNA is shown to be downregulated by *E. foeminea.* Beta-catenin is regulated by the Wnt signaling cascade in which Wnt glycoproteins bind both Frizzled receptors and LRP 5/6 coreceptors starting a cascade which through *disheveled* ultimately leads to phosphorylation of the beta-catenin destruction complex. Phosphorylation marks beta-catenin for degradation and vice versa; unphosphorylated beta-catenin migrates to the nucleus and acts as a coactivator, promoting the transcription of several downstream oncogenes such as c-Myc and cyclin-D1. Accordingly, aberrant, Wnt signaling significantly contributes to the development of several forms of cancer, including osteosarcoma. Studies have further shown that beta-catenin is relatively highly expressed in osteosarcoma [5].

RUNX2, another marker involved in osteosarcoma, was examined. RUNX2 is a transcription factor that is a primary regulator of both osteogenic differentiation and proliferation. As a controller of both differentiation and growth, RUNX2 expression has been shown to inhibit the proliferation of both normal osteoprogenitor and preosteoblast cells at the late G1 phase of the cell cycle. On the other hand, however, reports revealed that RUNX2 equally promotes cell proliferation in the early G1 phase [6]. RUNX2's promotion or inhibition of cell proliferation has thus been shown to be cell type-specific; whereas RUNX2 mainly inhibits growth in both osteoprogenitor and preosteoblast cells, it has been shown to promote cell proliferation in chondrocytes of RUNX2 null mice [7]. Further still, aortic endothelial cells showed increased proliferation when RUNX2 was ectopically expressed [8]. Such a dual functionality of RUNX2 thus suggests that RUNX2 protein could act as either a tumor suppressor or an oncoprotein. Osteosarcoma biopsies having high RUNX2 expression have been shown to have adverse tumor characteristics such as greater tumorigenicity, tumor progression, and metastases and have further been shown to regulate several cancer-related genes [6].

Key to the success of cancer drug therapies is their ability to induce programmed cell death in cancer cells. The effect of *E. foeminea* extracts on genes involved in apoptosis was thus assessed. BAX and p53 are two genes that play critical roles in regulating apoptosis. Most drugs that induce apoptosis do so via the intrinsic apoptotic pathway in which apoptosis is initiated by intracellular signals that ultimately lead to the opening of the mitochondrial inner membrane, loss of potential at the mitochondrial transmembrane, and finally release of proapoptotic proteins into the cytoplasm. These mitochondrial changes are regulated by the Bcl-2 protein family, which is composed of both pro- and antiapoptotic members [9]. BAX belongs to the proapoptotic group of the Bcl-2 family. Reports suggest that the expression of Puma, a Bcl-2 family member, is induced by p53 and that its overexpression most probably mediates BAX expression, structural change, and mitochondrial translocation, in addition to both the release of cytochrome c and mitochondrial membrane potential reduction. Other reports have further shown that p53 can directly activate BAX and initiate apoptosis [10].

Bioactive compounds are defined as compounds that exert either a positive or negative physiological effect on aliving system. Plant-derived compounds have been reported to alleviate several medical conditions, including inflammation, osteoporosis, and neurodegenerative diseases, in addition to a variety of chronic illnesses such as hypertension, diabetes mellitus, and cancer [11–13]. Medicinal plants are, therefore, a potential source of diverse and complex compounds such as flavonoids, phenols, alkaloids, lectins, and terpenes, which may possess probable anticancer properties [14]. Broadly, the anticancer activity of herbal medicines may be classified into two groups depending on their mode of action. These include immunomodulatory and chemopreventive plants. While immunomodulatory herbs boost the immune system's capability to recognize and eliminate tumor cells, chemopreventive herbal medicines on the other hand utilize complex bioactive compounds to interfere with tumor development via several mechanisms including apoptosis induction, inhibition of nucleic acid synthesis, topoisomerase inhibition, microtubule interference, and cell cycle alteration [14]. As of 2014, 90 out of 121 drugs approved for cancer treatment were associated with herbal plants. To add, studies show that between 1981 and 2002, 48 out of 65 newly registered cancer drugs were obtained from natural products, of which 25% were associated with medicinal plants [15].

*Ephedra foeminea* Forssk. (Ephedraceae) is a small evergreen climbing or hanging shrub, distributed in the Eastern Mediterranean region, mostly in montane zones [16]. The plant has tiny scale-like leaves (2.0 mm) with nonsucculent, spineless stems. Plants in the genus have been utilized as a source of *Ephedra* alkaloids that act as stimulants, weight loss agents, and dietary supplements. *Ephedra* alkaloids have however been reported to be associated with psychosis, severe depression, agitation, hallucinations, sleep disturbance, suicidal ideation, and several addiction symptoms [17]. The alkaloids have also been shown to cause stroke and heart problems, prompting a ban in the United States of drugs containing Ephedra, in 2004. Ephedra alkaloids were however not detected in *E. foeminea* [18].

Although little is known about the use of *E. foeminea* to treat cancer, its use among Middle Eastern patients recently became popular due to the belief that the herb possesses curative properties against cancer [19]. Ben-Arye and colleagues reported that the boiled water extract of *E. foeminea* showed no significant effect on MDA-MB231 and SKBR3 breast cancer cell lines [19]. However, another study [20] showed that *E. foeminea* ethanol, water, and fruit juice extracts reduced the percentage viability of these cell lines to varying degrees. Further still, the study reported similar observations when A549 lung carcinoma and HCT116 colorectal carcinoma cells were treated with *E. foeminea* herbal extracts [20]. The discrepancy in the results obtained by these studies thus warrants further investigation into *E. foeminea*'s claimed anticancer properties.

This study hypothesized that *E. foeminea* ethyl acetate, ethanol, and water extracts significantly affect U2OS cell viability and migration and the expression of genes involved in each of these processes. The main aim of this study was to assess the effect of *E. foeminea* ethyl acetate, ethanol, and water extracts on morphology, viability, migration, and gene expression of U2OS osteosarcoma cells.

The first step implemented in the isolation of bioactive compounds is extraction. The yield of bioactive natural products tends to be relatively low and is greatly affected by the extraction method of choice [21]. The type of extraction method employed may further affect the chemical properties of resulting extracts [22]. Based on the extraction principle, methods of extraction include distillation, pressing, sublimation, and solvent extraction; among these, solvent extraction is the most commonly used method. Its extraction efficiency may be affected by the extraction-solvent properties, raw material particle size, solvent ratio to solid, temperature, and duration of extraction [21]. Subsequently, this study utilized a method of solvent extraction called maceration for crude extract preparation. Maceration involves soaking the ground plant material in a solvent and letting the mixture stand at room temperature with frequent agitation until all soluble matter is dissolved; this is followed by pressing the solid residue and filtering the total resulting liquid [23]. Although maceration may be time-consuming, it is a simple procedure and is suitable for extracting thermolabile compounds [21].

In this study, *E. foeminea* crude extracts were prepared using ethyl acetate (EtOAc), ethanol (EtOH), and water (H_2_O) as solvents. The effect of each extract on the viability of U2OS osteosarcoma cells was determined using MTT assay while the wound healing assay was employed to study the extracts' effect on U2OS cell migration ability. Differential gene expression of several markers such as *beta-catenin* (*B-catenin*), *Twist-1*, and *RUNX2* that regulate key pathways involved in both cell proliferation and migration was tested. All extracts tested in this study significantly reduced U2OS cell viability in a manner dependent on both dose and time. In addition, the ethanol extract significantly reduced U2OS migration ability. All extracts downregulated the steady-state mRNA expression of several genes involved in both cell proliferation and migration, with statistically significant effects observed for both ethanol and water extracts. The hemolysis assay employed to test *E. foeminea* extract hemolytic activity showed a negligible effect of both ethanol and water extracts on erythrocyte lysis. These results suggest that *E. foeminea* crude extracts most probably affect U2OS proliferation and migration ability by modulating key genes involved in the regulation of these processes and possess no cytotoxicity to human erythrocytes [24].

## 2. Materials and Methods

### 2.1. Plant Material Harvest

Plant aerial parts (the scale minute leaves and stems) of *E. foeminea* were collected from a female plant in July at the flowering stage from Hebron city; Lat: 31.538629, Lon: 35.085769. A voucher specimen was preserved for identification in the Palestine-Korea Biotechnology Center at the Palestine Polytechnic University. Morphological identification of the plant was carried out by referring to the Flora of Israel Online by Avinoam Danin (http://flora.org.il/en/plants/ephfoe/).

### 2.2. Crude Extract Preparation

The collected plant material was shade dried at room temperature then cut into small pieces of 1.0 to 2.0 cm and ground with an electric grinder into a fine powder. After sieving, the powder was separated from the residual fibers; then, one gram of the powder was mixed with 30.0 mL of the following analytical grade solvents, deionized water, absolute ethanol, and ethyl acetate. After 24 h of continuous shaking (170 rpm), the mixture was filtered, and the filtrate evaporated in a fume hood until entirely dried. The dry extract yield as a percentage of the starting powder was calculated and found to be 2%, 6.8%, and 21.2% for ethyl acetate, ethanol, and water extracts, respectively. The dried extracts were dissolved in DMSO to make stock solutions of 150 mg/mL; from these stock solutions, a series of working concentrations (0.5, 0.25, 0.13, 0.06, and 0.03 mg/mL) were prepared by serial dilution using complete Dulbecco's modified Eagle's medium (DMEM) cell culture media.

### 2.3. Cell Culture

The cell line used for this study was the U2OS human osteosarcoma cell line (kindly provided by Dr. Andreas Kakarougkas, Department of Biology, The American University in Cairo). Cells were cultured in DMEM basal media (Invitrogen, USA), supplemented with 10% fetal bovine serum (FBS) (Invitrogen) and 5% Pen-Strep from Invitrogen. Cells were cultured at 37°C and 5% CO_2_ in a humidified incubator.

### 2.4. U2OS Doubling Time

U2OS doubling time was determined by culturing U2OS cells in a 6-well plate over a period of 6 days. Viable cell counts were made on each consecutive day using trypan blue exclusion assay. At the end of this period, a growth curve of viable cell count against time was generated and doubling time determined using equation (1) obtained from the American Type Culture Collection (ATCC) website [25]:

(1) $$\begin{array}{c} \text{DT}=T\frac{\ln2}{\ln\;\left(X_{\text{e}}/X_{\text{b}}\right)}, \end{array}$$

where DT is the doubling time. *T* is the total time during which exponential growth occurs. *X*_b_ is the cell number at the beginning of exponential growth. *X*_e_ is the cell number at the end of the exponential growth.

### 2.5. MTT Cell Viability Assay

The viability of U2OS cells incubated with *E. foeminea* extracts for 24 h, 48 h, and 72 h was determined using the MTT viability assay. Viable cells reduce MTT reagent (3-(4,5-dimethylthiazolyl-2)-2,5-diphenyltetrazolium bromide) (Serva, Germany) to form a purple color by mitochondrial dehydrogenase enzymes [26]. U2OS cells were incubated with *E. foeminea* extracts in a 96-well plate for 24 h, 48 h, or 72 h. The culture media was aspirated and 100 *μ*L of fresh media containing 20 *μ*L of MTT solution (5.0 mg/mL) was added per well and incubated for 4 h. The culture media was discarded after incubation and replaced with 100 *μ*L of dimethyl sulfoxide (DMSO) (Sigma-Aldrich, USA) per well to solubilize the purple formazan crystals formed as a result of MTT reduction. The color change was quantified by taking absorbance readings at 570 nm using SPECTROstar Nano microplate reader (BMG LABTECH). The percentage of cell viability was calculated by expressing the absorbance of the treated wells as a percentage of the untreated wells.

### 2.6. Scratch Wound Healing Assay

The scratch wound healing assay was performed to test the cell migration ability of U2OS cells when incubated with *E. foeminea* extracts. U2OS cells were seeded at a density of 3 × 10^5^ cells per well in a 6-well plate and incubated until cells were about 90% confluent. At this point, two perpendicular scratches were made in the cell monolayer with the help of a sterile 20 *μ*L pipette tip. Cells were then washed twice with 1x PBS to remove cell debris, before adding fresh complete DMEM containing extract treatments whose concentrations were adjusted to the least obtained IC_50_ per extract. A series of images along the scratch were taken at various points per treatment at 0 h. Cells were then incubated at 37°C/5% CO_2_ and consecutive images taken after 6 h and 12 h for similar points along the scratch as those previously taken at 0 h. All images were captured using the Olympus IX70 inverted microscope. Areas of the wounds were measured using ImageJ 1.51j8 software [27] and percentage wound closure determined using equation (2) [28]:

(2) $$\begin{array}{c} \text{WC}\%=\frac{\text{WC }0\text{ h}-\text{WC }X\text{ h}}{\text{WC }0\text{ h}}\times 100, \end{array}$$

where WC % is the percentage wound closure. WC 0 h is the percentage wound closure at zero hours. WC *X* h is the percentage wound closure at a specified time point.

### 2.7. Reverse Transcription Polymerase Chain Reaction (RT-PCR)

Analysis of differential gene expression between treated and untreated cells was performed using semiquantitative RT-PCR in which 0.5 *μ*g of total RNA was reverse transcribed using RevertAid First Strand cDNA Synthesis Kit (Thermo Scientific, USA) facilitated by random primers in a final volume of 20 *μ*L, according to the manufacturer's protocol. The PCR reaction was performed using 1 *μ*L cDNA template with the MyTaq DNA Polymerase Kit (Bioline) with GAPDH serving as an endogenous control. With the exception of cycle numbers and annealing temperatures (Table S1), PCR conditions were as follows among all tested genes: 94°C for 3 minutes, followed by cycles of 94°C for 30 seconds, annealing temperature for 30 seconds, and 72°C for 45 seconds with a final extension carried out at 72°C for 7 minutes. PCR products were analyzed on 2% agarose gel, and visualization was done using the Gel Doc EZ System (Bio-Rad, USA). Working solution concentrations for all primers used were set to 10 pmol with the exception of GAPDH primers whose working concentration was set to 5 pmol.

### 2.8. In Silico Analysis

U2OS microarray datasets in which U2OS cells were treated with either doxorubicin or nutlin-3 [29] were retrieved from the Gene Expression Omnibus (GEO) database. Microarray datasets were processed in R using Bioconductor package annaffy (http://www.bioconductor.org/packages/release/bioc/html/annaffy.html) [30]. The analysis was made for differentially expressed genes with both *P* value and fold change cutoffs, respectively, set to 0.05 and 2. Accession numbers and descriptions of the various datasets used are shown in Table S2.

### 2.9. Hemolysis Assay

After obtaining approval from the Institutional Review Board (IRB) of the American University in Cairo, blood samples from healthy volunteers were collected, after informed consent was obtained, by a physician at the university clinic. 2.0 mL of fresh blood was acquired per experiment. Human erythrocytes mixed with EDTA (ethylenediaminetetraacetic acid) as an anticoagulant were centrifuged for 5 minutes at 1000 g at 4°C, then washed twice in PBS for complete removal of serum. Red blood cells (RBCs) were finally diluted to 2% concentration in PBS, and 60 *μ*L of this solution was added to each well in a flat-bottomed 96-well plate. 60 *μ*L of either ethanol or water extract was added per well such that the final erythrocyte concentration was 1%. Concentrations close to IC_75_, IC_50_, and IC_25_ were tested per extract; these included 260.4 *μ*g/mL, 97.5 *μ*g/mL, and 36.5 *μ*g/mL for the ethanol extract along with 127.7 *μ*g/mL, 30.8 *μ*g/mL, and 7.4 *μ*g/mL for the water extract. Plates were incubated for 1 h at 37°C. Untreated RBCs and deionized water were, respectively, used as controls for 0% and 100% hemolysis. Plates were then centrifuged at 3000 g for 10 minutes at 4°C, after which 100 *μ*L of supernatant was transferred to a clean flat-bottomed plate. The supernatant's absorbance was measured at 570 nm and the percentage of hemolysis determined using equation (3) [31]:

(3) $$\begin{array}{c} \text{Hemolysis }\left(\%\right)=\frac{\left(A_{\max}-A_{\text{t}}\right)}{\left(A_{\max}-A_{\min}\right)}\times 100, \end{array}$$

where *A*_max_, *A*_min_, and *A*_t_ represent the absorbance values for untreated, completely hemolyzed, and tested RBCs, respectively.

### 2.10. Statistical Analysis

All data generated were presented as the mean ± standard deviation of three independent experiments unless otherwise specified and were analyzed using GraphPad Prism 6.01 software [32]. Multiple comparison analyses were performed using either two-way or one-way ANOVA (analysis of variance) followed by Dunnett's post multiple comparisons test. Pairwise analyses were, on the other hand, performed using GraphPad Prism's multiple *t*-test analysis with multiple comparisons corrected for using the Holm-Sidak method.

Dose-response curves and IC_50_ values were generated in GraphPad Prism 6.01 using (log(inhibitor) vs.normalized response − variable slope). ImageJ software was used to analyze the intensities of the PCR bands; these were normalized to the endogenous control (GAPDH) and used to calculate relative gene expression presented as fold change relative to untreated cells. *P* values less than 0.05 were considered significant (∗*P* < 0.05, ∗∗*P* < 0.01, and ∗∗∗*P* < 0.001).

## 3. Results

### 3.1. U2OS Growth Characteristics

U2OS cells used in this study were cultured over a period of six days, and the number of viable cells per mL was determined for each consecutive day. The obtained growth curve exhibited a sigmoidal pattern with lag, exponential, and stationary phases during which cells exhibited slow, exponential, and stationary growth rates, respectively. The lag phase lasted 2 days, after which the cells grew exponentially until the fourth day. The cell growth rate then decelerated between the 4th and 5th days and finally leveled off on the 6th day. The number of cells increased by about sevenfold during the entire growth period with a calculated doubling time of 1.2 days (Figure S1).

### 3.2. MTT Assay

**Figure 1:**
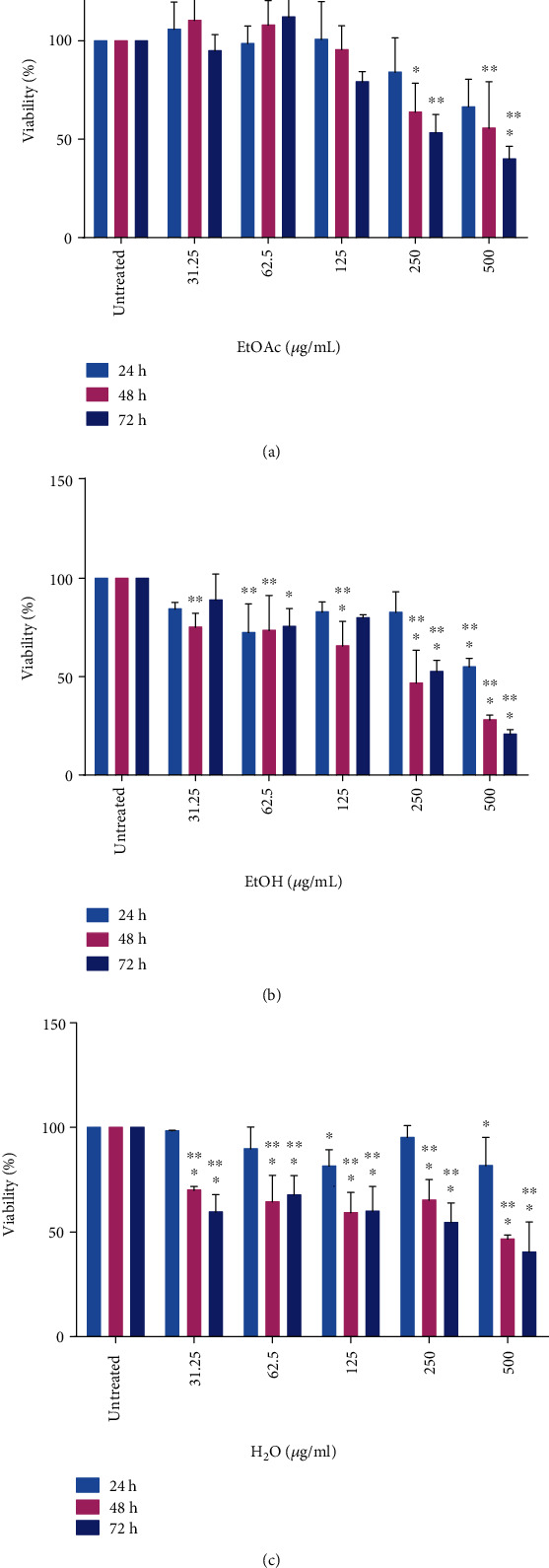
Effect of *E. foeminea* (a) ethyl acetate, (b) ethanol, and (c) water extracts on U2OS cell viability; all extracts generally showed a reduction in percentage cell viability that was both dose- and time-dependent. ∗∗∗*P* ≤ 0.001, ∗∗*P* ≤ 0.01, and ∗*P* ≤ 0.05; *n* = 3.

MTT assay was utilized to determine the effect of the various *E. foeminea* extracts on U2OS cell viability over a series of concentrations ranging from 31.25 *μ*g/mL to 1000 *μ*g/mL at 24 h, 48 h, and 72 h time points. Generally, when treated with *E. foeminea* ethyl acetate extract, U2OS cells showed a reduction in percentage cell viability that was both dose- and time-dependent (Figure 1(a)). Percentage viability reduced with increasing extract concentration; significant reductions in viability were observed at concentrations of 250 and 500 *μ*g/mL for both the 48 h and 72 h time points when compared to untreated cells. The reduction in cell viability at the 24 h time point showed no statistical significance.

All concentrations below 250 *μ*g/mL showed no significant decrease in viability. Furthermore, percentage viability declined with increasing incubation time for individual extract concentrations. The most significant reduction in cell viability was observed at the extract concentration of 500 *μ*g/mL for the 72 h time point (∗∗∗*P* < 0.001) while the least significant reduction in cell viability was observed at 250 *μ*g/mL for the 48 h time point.

The *E. foeminea* ethanol extract showed a similar trend to the ethyl acetate extract with regard to its effect on U2OS cell viability (Figure 1(b)). The 500 *μ*g/mL concentration significantly decreased the viability of U2OS cells at all time points with *P* values of less than 0.0001. The ethanol extract at 250 *μ*g/mL significantly reduced the viability of U2OS cells at only 48 h and 72 h time points (∗∗∗*P* < 0.001). Other significant decreases in viability were observed at extract concentrations of 125 *μ*g/mL for both 48 h and 72 h time points (*P* values 0.001 and 0.02, respectively). In addition, the extract concentration at 62.5 *μ*g/mL also showed significant decreases in viability at 24 h, 48 h, and 72 h time points (*P* values 0.0026, 0.0020, and 0.0065, respectively). The least significant reduction in viability was observed for the 31.25 *μ*g/mL ethanol extract concentration at only the 48 h time point (*P* value 0.002). The greatest decline in U2OS cell viability was observed for the ethanol extract with a concentration of 500 *μ*g/mL at the 72 h time point.

U2OS cells treated with *E. foeminea* water extract similarly showed a decline in viability in a manner that was both dose- and time-dependent (Figure 1(c)). All concentrations of the water extract showed significant decreases in viability at all time points except the extracts at 250, 62.5, and 31.25 *μ*g/mL for the 24 h time point. Extract concentrations that showed the highest significant decrease in percentage viability (∗∗∗*P* < 0.001) included 62.5, 125, 250, and 500 *μ*g/mL for both the 48 h and 72 h time points.

**Figure 2:**
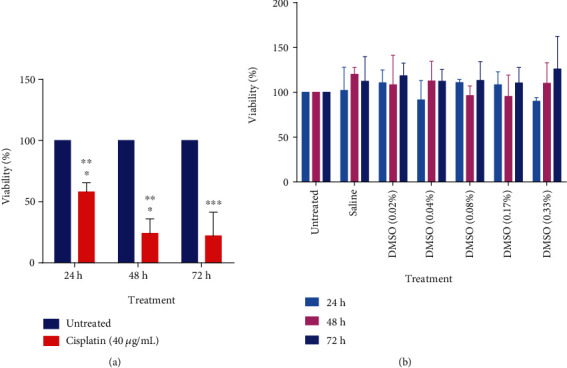
Effect of cisplatin and DMSO on U2OS viability. (a) The positive control, cisplatin (40 *μ*g/mL), significantly reduced U2OS viability at all examined time points. (b) DMSO, in which *E. foeminea* extracts were dissolved, appeared to have no significant effect on U2OS cell viability. ∗∗∗*P* ≤ 0.001, ∗∗*P* ≤ 0.01, and ∗*P* ≤ 0.05; *n* = 3.

Cisplatin (40 *μ*g/mL), used as a positive control, showed significant decreases in U2OS viability at all time points tested (Figure 2(a)). DMSO, in which extracts were dissolved generally, had no significant effect on U2OS cell viability (Figure 2(b)) at all concentrations for all time points. *P* values obtained for all extract treatments are summarized in Table S3.

### 3.3. IC_50_

**Figure 3:**
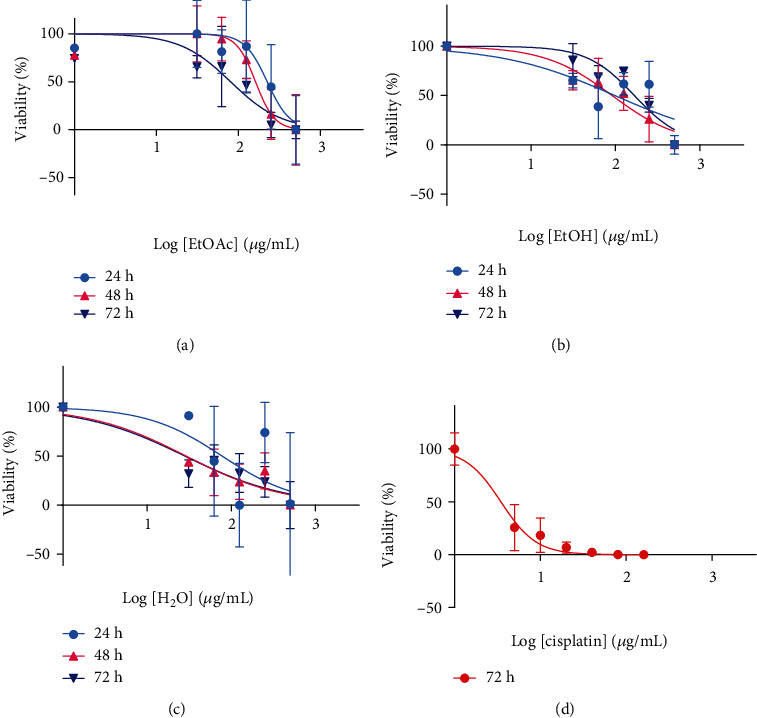
U2OS dose-response curves. *E. foeminea* (a) ethyl acetate, (b) ethanol, and (c) water extracts. (d) Dose-response curve for cisplatin (positive control); percentage viabilities were normalized to a scale running from 0% to 100%.

Results obtained from the MTT assay were used to determine the IC_50_ values for all extracts at all time points using nonlinear regression analysis. Dose-response curves (Figures 3(a)–3(c)) for *E. foeminea* ethyl acetate, ethanol, and water extracts were plotted for 24 h, 48 h, and 72 h time points and used to generate IC_50_ values per extract per time point (Table 1). The least IC_50_ values obtained per extract were 80.4 *μ*g/mL at 72 h, 91.2 *μ*g/mL at 48 h, and 28.23 *μ*g/mL at 72 h, respectively, for ethyl acetate, ethanol, and water extracts.

Concentrations close to the least obtained IC_50_ per extract were used for downstream analyses; these concentrations included 80.353 *μ*g/mL, 97.499 *μ*g/mL, and 30.761 *μ*g/mL for ethyl acetate, ethanol, and water extracts. The obtained extract IC_50_ values were compared to the IC_50_ of cisplatin when incubated with U2OS cells for 72 h. The effect of varying concentrations of cisplatin ranging from 5 to 160 *μ*g/mL on the viability of U2OS cells was studied; the generated dose-response curve (Figure 3(d)) was utilized to calculate the IC_50_ of cisplatin at the 72 h time point (Table 1). Cisplatin IC_50_ (3.473 *μ*g/mL) was over 18 times lower than that of all *E. foeminea* extracts.

Obtained Hill-slope coefficients for cisplatin (at 72 h), ethyl acetate (at 72 h), ethanol (at 48 h), and water (at 72 h) extracts were compared; apart from the water extract, all other treatments possessed Hill-slope coefficients greater than 1, with the highest ratio observed for cisplatin. The least obtained IC_50_ per treatment also possessed relatively low Sy.x (standard deviation of the residuals) values, indicating that the goodness of fit for the data collected at these time points was acceptable (Table 1).

### 3.4. Cell Morphology

The effect of *E. foeminea* extracts on U2OS cell morphology was studied. Ethyl acetate, ethanol, and water extract treatments, respectively, adjusted to concentrations close to the least obtained IC_50_ value per treatment. *E. foeminea* extracts showed no marked effect on cell morphology when compared to untreated cells at all time points. Generally, U2OS cells used for this study grew as a monolayer adherent to the surface of the culture flasks. The cells mostly possessed an elliptical shape in which they appeared wider at the center but tapered on either end (Figure S2).

### 3.5. Wound Healing Assay

**Figure 4:**
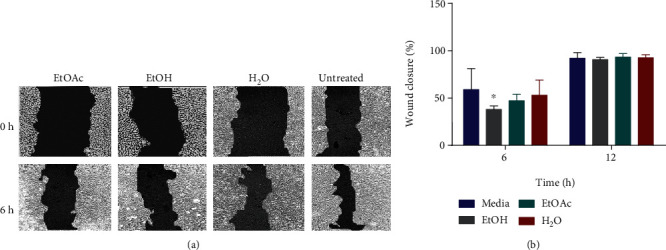
Effect of *E. foeminea* on U2OS cell migration. (a) Wound healing assay representative images at the 6 h time point. (b) Percentage wound closure measured at both 6 h and 12 h time points. All extracts reduced U2OS percentage wound closure at the 6 h time point with only the ethanol extract showing statistically significant results; ∗*P* = 0.013, *n* = 3.

The wound healing assay was performed, and the percentage wound closure was determined for both 6 h and 12 h time points. At the 6 h time point, results of percentage wound closure showed that untreated cells had the highest wound closure, respectively, followed by cells treated with water, ethyl acetate, and ethanol extracts. At this time point, percentage wound closure for all cells treated with extract was lower than that of untreated cells, although a significant decline in percentage wound closure was observed for only cells treated with ethanol extract (*P* value = 0.013). There was no significant difference in percentage wound closure at the 12 h time point when treated cells were compared to untreated ones (Figure 4).

### 3.6. RT-PCR

**Figure 5:**
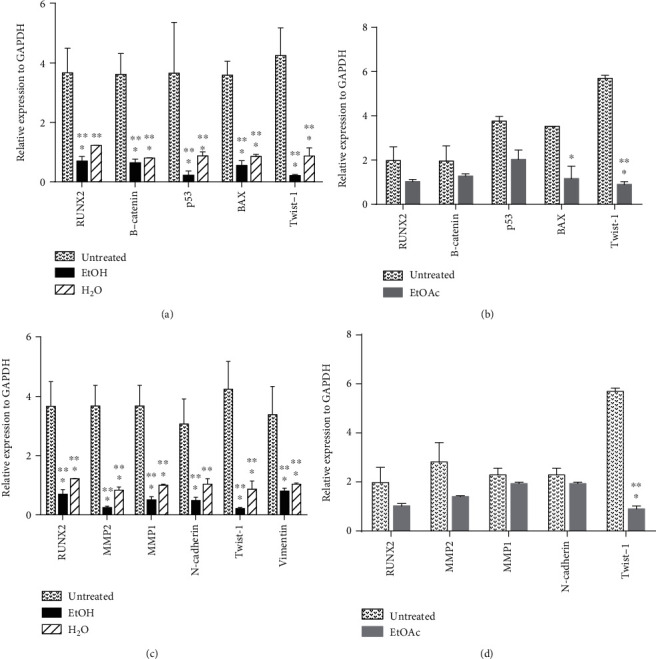
RT-PCR ImageJ analysis. (a, b) mRNA steady-state levels for genes (RUNX2, B-catenin, p53, BAX, and Twist-1) involved in cell proliferation and apoptosis. (c, d) mRNA steady-state levels for genes (*RUNX2*, *MMP2*, *MMP1*, *N-cadherin*, *Twist-1*, and *vimentin*) involved in cell migration. U2OS cells were treated with either ethanol, water, or ethyl acetate extracts adjusted to the least IC_50_ value obtained per extract at respective time points. ∗∗∗*P* ≤ 0.001, ∗∗*P* ≤ 0.01, and ∗*P* ≤ 0.05; *n* = 3.

mRNA expression for various genes involved in either cell proliferation and apoptosis or cell migration was assessed in both treated and untreated cells using RT-PCR (Figure 5) (Figure S3). mRNA steady-state levels for several genes involved in either cell proliferation and apoptosis (RUNX2, B-catenin, p53, BAX, and Twist-1) (Figures 5(a) and 5(b)) or cell migration (RUNX2, MMP2, MMP1, N-cadherin, Twist-1, and vimentin) (Figures 5(c) and 5(d)) were assessed for U2OS cells treated with either ethanol, water, or ethyl acetate extracts adjusted to the least IC_50_ value obtained per extract. The expression of all genes tested was significantly decreased by varying folds in both the ethanol and water extracts. The ethanol extract reduced the expression of the tested genes much more than the water extract. The genes whose expression was reduced the most by the ethanol extract included Twist-1, p53, and MMP2 while B-catenin, RUNX2, and vimentin were the least affected. A similar trend was observed for the ethyl acetate extract in which all tested genes were downregulated. However, apart from Twist-1, the downregulation of all other tested genes by the ethyl acetate extract showed no statistical significance when compared to the untreated cells (Table 2).

### 3.7. Comparison of U2OS Gene Expression when Treated with Either *E. foeminea* Extracts, Doxorubicin, Or Nutlin-3

**Figure 6:**
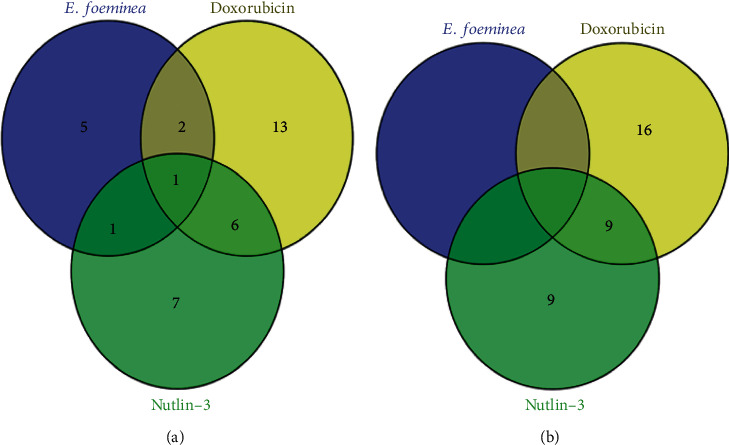
Venn diagrams comparing gene expression in U2OS cells treated with either *E. foeminea*, doxorubicin, or nutlin-3. (a) represents the number of downregulated genes while (b) represents the number of upregulated genes per treatment.

U2OS gene expression datasets for both doxorubicin and nutlin-3 treatments were obtained from the GEO database and analyzed for differentially expressed genes at a *P* value cutoff of 0.05 and fold cutoff of 2; the comparison was made to U2OS gene expression when treated with *E. foeminea* extracts. Of the 9 genes examined in this study, 5 (BAX, MMP1, MMP2, TP53, and vimentin) were exclusively downregulated by *E. foeminea* crude extracts while 2 (B-catenin and N-cadherin) were downregulated in both *E. foeminea* and doxorubicin treatments only. On the other hand, only 1 (Twist-1) gene was downregulated solely by both *E. foeminea* and Nutlin-3 while all the three treatments were shown to downregulate 1 (RUNX2) gene. Although *E. foeminea* showed no upregulation of any of the tested genes, BAX was upregulated by both doxorubicin and nutlin-3 treatments while Twist-1 was exclusively upregulated by doxorubicin (Figure 6, Table 3). The entire lists of all genes analyzed for both doxorubicin and nutlin-3 treatments are, respectively, presented in Supplementary Files 1 and 2. Both logFC (log of fold change) and *P* values for each gene are reported.

### 3.8. Hemolysis Assay

**Figure 7:**
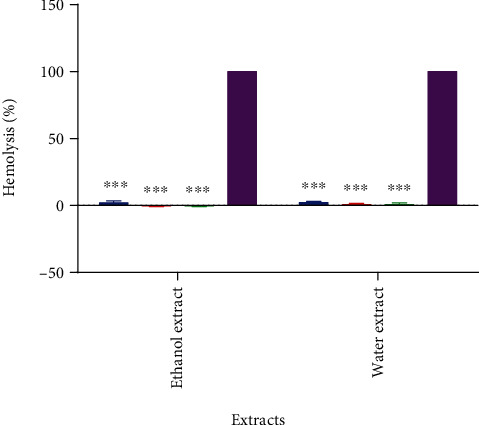
Hemolytic activity of *E. foeminea* ethanol and water extracts. Deionized water (purple) represents 100% hemolysis. Three concentrations for each extract were tested: 260.4 *μ*g/mL (blue), 97.5 *μ*g/mL (red), and 36.5 *μ*g/mL (green) for the ethanol extract along with 127.7 *μ*g/mL (blue), 30.8 *μ*g/mL (red), and 7.4 *μ*g/mL (green) for the water extract. ∗∗∗*P* ≤ 0.001, *n* = 3.

Hemolytic activity for both ethanol and water extracts was tested on human erythrocytes using the hemolytic assay. Since the ethyl acetate extract gave the least biological activity, it was excluded from the hemolysis test. The hemolytic assay measures the amount of hemoglobin liberated from lysed RBCs by spectrophotometric means [33]. Hemolytic activities for both ethanol and water extracts adjusted to concentrations close to IC_75_, IC_50_, and IC_25_ were compared to both untreated RBCs (no hemolysis) and RBCs treated with deionized water (100% hemolysis). All tested concentrations for both extracts showed negligible hemolytic activity, comparable to the untreated RBCs. The ethanol extract generally showed lower hemolytic activity than the water extract at all tested concentrations; however, this difference showed no statistical significance (Figure 7).

## 4. Discussion

The obtained growth curve for U2OS cells used in this study showed definite lag, exponential, and stationary phases during which cells, respectively, went through a period of slow growth, followed by a period of exponential growth and finally transitioned into another slow growth phase as cells reached confluency [34] (Figure S1). U2OS cell growth with respect to this study followed a characteristic sigmoid growth pattern with a doubling time of 1.2 days. This was similar to the doubling time of 1.25 days previously reported by Rey and colleagues [35]. However, U2OS doubling times as high as 2.75 days have been reported [36].

The effect of *E. foeminea* ethyl acetate, ethanol, and water extracts on U2OS cell viability was determined using MTT assay, which involves assessing the metabolic activity of cells by quantifying the intensity of purple formazan crystals formed after viable cells reduce the yellow MTT reagent [37]. *E. foeminea* ethyl acetate, ethanol, and water extracts each significantly decreased U2OS percentage cell viability in a manner dependent on both concentration and time (Figures 1(a)–1(c)); generally, longer incubation times and higher extract concentrations favored the reduction of U2OS percentage cell viability. These results are in agreement with previous findings where both *E. foeminea* ethanol and fruit juice extracts significantly decreased the viability of MDA MB 231 breast cancer, HCT 116 colorectal cancer, and normal HaCat keratinocyte cells [20]. Our results however contradicted with those of Ben-Arye and colleagues in which they reported that *E. foeminea* boiled water extract showed no significant effect on the percentage viability of both MDA MB 231 and SKBR3 breast cancer cells [19]. This disparity can be attributed to the difference in extraction methods employed per study; it has been shown that different extraction procedures have varying influences on the physical, chemical, and biological properties of the final extract [38]. DMSO, in which our extracts were dissolved, showed no significant effect on U2OS cell viability (Figure 2(b)), indicating that the working concentrations of DMSO present in the prepared extracts did not significantly interfere with U2OS viability.

Based on results obtained from the MTT assay, dose-response curves were plotted for all *E. foeminea* extracts tested at 24 h, 48 h, and 72 h time points (Figures 3(a)–3(c)) and used to determine IC_50_ values per extract per time point (Table 1). When IC_50_ values per extract were compared at each time point, results showed that the ethyl acetate extract IC_50_ decreased at each consecutive time point with the 24 h time point as a reference. On the other hand, IC_50_ values of both ethyl acetate and water extracts were at their lowest at the 72 h time point but slightly higher at the 48 h time point. These results suggest that the potency of *E. foeminea* extracts is influenced by the incubation time. While *E. foeminea* ethanol extract was most potent at the 48 h time point, both the ethyl acetate and water extracts showed the greatest potency at the 72 h time point. For both the ethanol and water extracts, a slight reduction in cytotoxicity is observed after 72 h of incubating the U2OS cells with the respective extract. This result could be related to the doubling time of the U2OS cells. After 72 h, the surviving cells would have tripled, producing new “unaffected” cells. All *E. foeminea* IC_50_ values obtained in this study were relatively lower than those previously reported [20]; these were about 570 *μ*g/mL and 750 *μ*g/mL, respectively, for MDA-MB-231 and HaCat cells treated with *E. foeminea* ethanol extract for 48 h. This suggests that in addition to the extraction method employed, the potency of *E. foeminea* probably varies from one cell line to another. A comparison of the least obtained IC_50_ values per extract shows that the least IC_50_ values were observed in the most polar extraction solvent which was water than in the moderately polar ethanol and ethyl acetate solvents [39]. It is likely that the more polar fractions of *E. foeminea* contain higher amounts of the active inhibitory compound(s). Cisplatin, a drug commercially used in osteosarcoma treatment [40], had a much lower IC_50_ than all IC_50_ values obtained for all *E. foeminea* extracts at all time points (Figure 3(d), Table 1). The high *E. foeminea* extract IC_50_ values relative to cisplatin can be partly explained by the fact that the *E. foeminea* extracts were tested in their crude form without purifying the active compound(s) [41].

Hill coefficients for all extract treatments were generally above 1, with the exception of the water extract (Table 1). For biological reactions, the Hill coefficient is a parameter used to reflect the steepness of dose-response curves; the higher the Hill coefficient, the steeper the curvature and vice versa. It has been shown that Hill coefficients could further give an idea of the number of either receptors or ligands involved in inhibitor binding. A Hill coefficient of 1 is indicative of a response elicited by the binding of a single ligand to a single receptor, while Hill coefficients greater than 1 could indicate the involvement of more than one ligand in binding a receptor or group of receptors [42]. Hill coefficient results from this study thus suggest that there could be a greater variety of ligand-receptor interactions in both the ethyl acetate and ethanol extracts than in the ethanol extract. It should, however, be noted that although the Hill coefficient can serve to reflect the number of ligands or receptors involved in inhibition, it does not indicate the type of binding (either competitive, noncompetitive, ortho-, iso-, or allosteric) involved [42].

Cell migration is fundamental to promoting tumor progression and metastasis [43]. Results from this study show that all extract treatments reduced U2OS percentage wound closure with a statistically significant decrease observed for only the ethanol extract treatment (Figure 4). This shows that *E. foeminea* extracts reduced the rate by which U2OS cells migrated to close the wound created by the scratch at the beginning of the assay. It is thus probable that *E. foeminea* extracts negatively influence the molecular machinery involved in U2OS cell migration.

In cancer, bioactive natural compounds have been shown to affect gene expression through modulating key transcription factors and pathways involved in cancer pathology [44]. In osteosarcoma, one such pathway is the Wnt/B-catenin signaling pathway which enhances the transcription of several downstream Wnt-responsive genes such as those involved in the remodeling of the extracellular matrix and in cell cycle regulation when B-catenin is translocated to the nucleus [45]. In this study, *E. foeminea* extracts downregulated *B-catenin* steady-state mRNA expression (Figures 4(a), 4(b), and S3 and Table 2), suggesting a decline in the amounts of B-catenin protein and thus a subsequent decrease in the expression of downstream WNT-responsive genes. A number of studies have previously shown that several compounds of natural origin can modulate the Wnt/B-catenin signaling pathway. Curcumin was shown to suppress Wnt/B-catenin signaling by activating both *Oct4* and glycogen synthase kinase 3B (GSK-3B) in both human embryonic kidney 293T cells and NCCIT human embryonic carcinoma cells. Further still, curcumin mitigated cell invasion, migration, and proliferation in U2OS, SaOS-2, and HOS osteosarcoma cells via Wnt/B-catenin suppression [44].

Both *Twist-1* and *RUNX2* expression is activated by the Wnt/B-catenin signaling pathway [46, 47]. Triggered expression of *RUNX2* by Wnt/B-catenin signaling was shown to promote the expression of several genes such as *MMP1* and *MMP2* that promote cell migration and metastasis in osteosarcoma by remodeling the extracellular matrix [46, 48]. In addition, *Twist-1* activation has been implicated in many cancers in which it promotes the expression of several genes involved in angiogenesis chemoresistance, stemness, and metastasis [47]. *Twist-1* has been shown to promote the expression of *both N-cadherin* and *vimentin* whose expression has been associated with increased migratory and invasive ability in several cancers. It is thus possible that the downregulation of B-catenin by *E. foeminea*, in turn, leads to decreased expression of both *Twist-1* and *RUNX2* transcription factors which subsequently reduces the expression of their downstream targets that in reference to this study include *N-cadherin*, *vimentin*, *MMP1*, and *MMP2* (Figure 5, Figure S3, and Table 2).

**Figure 8:**
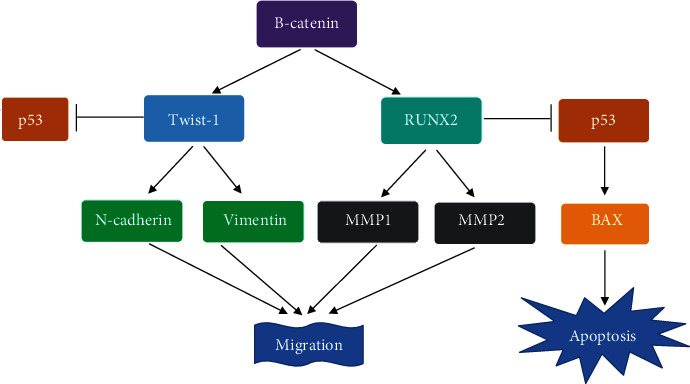
Depicted effect of *E. foeminea* extracts on U2OS gene expression. *E. foeminea* downregulated the steady-state mRNA expression of markers involved in cell proliferation, migration, and apoptosis. By downregulating the steady-state mRNA expression of both *B-catenin* and *p53*, downstream markers of cell proliferation, migration, and apoptosis are, respectively, downregulated.

*E. foeminea* treatments downregulated proapoptotic markers *p53* and *BAX* as well (Figure 5, Figure S3). Since both *Twist-1* and *RUNX2* are inhibitors of p53-mediated apoptosis [5, 47], it was expected that their downregulation would cause an upregulation of p53. This was not the case, however, since *p53* expression was shown to be downregulated in *E. foeminea* treatments. It is thus probable that in addition to targeting the Wnt/B-catenin pathway, *E. foeminea* independently targets the p53-mediated apoptotic pathway by downregulating *p53* and subsequently downregulating *BAX*. A schematic representation of the proposed path by which *E. foeminea* crude extract treatments downregulate migration markers (*MMP1*, *MMP2*, *N-cadherin*, and *vimentin*) along with proapoptotic markers (*p53* and *BAX*) is shown in Figure 8. Effect on gene expression is mostly dependent on the lipophilicity of a ligand, which determines whether the ligand directly crosses the cell membrane or instead binds to a transmembrane receptor and hence affects signal transduction cascades [49]. The more polar extracts such as water and ethanol probably contain more lipophobic ligands that likely bind to membrane receptor(s), most probably a G protein-coupled receptor or a receptor tyrosine kinase [50].

It is not uncommon for chemotherapeutic drugs to modulate gene expression [51–53]; doxorubicin, a standard chemotherapeutic drug against osteosarcoma [54], is a nonspecific drug that stops replication by preventing DNA cut by topoisomerase II from being resealed. It may also increase free radical production, hence adding to its cytotoxicity. Finally, it is also known to intercalate between base pairs, inducing histone eviction from transcriptionally active chromatin [54]. Nutlin-3 is an imidazoline analog that disrupts the interaction between p53 and MDM2, resulting in a growth inhibition state called senescence. Nutlin-3 works best on tumors that contain wild-type p53 and has been shown to exert its effect within minutes after treatment [55].

U2OS gene expression when treated with either *E. foeminea*, doxorubicin, or nutlin-3 was assessed (Figure 6, Table 3). Only one gene (*RUNX2*) out of nine was significantly downregulated by all the three treatments; it is possible that *RUNX2* could be a potent therapeutic target in osteosarcoma treatment [46]. Both *B-catenin* and *N-cadherin* were downregulated by *E. foeminea* and doxorubicin but not nutlin-3; *Twist-1* was however downregulated by both *E. foeminea* and nutlin-3 but not doxorubicin. It is also worth noting that both *MMP1* and *MMP2* were exclusively downregulated by *E. foeminea*, indicating that *E. foeminea* probably reduces extracellular matrix remodeling by downregulating MMPs and thus decreasing cell migration.

The ability of *E. foeminea* extracts to lyse normal human erythrocytes was negligible (Figure 7). It has been reported that several medicinal plants contain chemical compounds that could promote hemolysis and result in adverse conditions such as hemolytic anemia. It is thus critical that medicinal plants are tested for possible hemolytic potential [56]. Our results indicate that *E. foeminea* extracts exhibit minute hemolytic activity and are likely cytocompatible with human erythrocytes even at concentrations greater than IC_50_ [57].

## 5. Conclusions

Taken together, this study shows that *E. foeminea* extracts significantly reduce U2OS cell viability and migration. All extracts downregulated the steady-state mRNA expression of key markers involved in regulating these processes. Finally, *E. foeminea* extracts exhibited negligible hemolytic activity.

It seems clear that *E. foeminea* ethyl acetate, ethanol, and water extracts contain different phytoconstituents with different cytotoxicities; chemical profiling, analysis of active extracts, and identification of compounds using methods such as high-performance liquid chromatography (HPLC), liquid chromatography-mass spectrometry (LC-MS), and/or gas chromatography-mass spectroscopy (GCMS) are recommended.

## Figures and Tables

**Table 1:** Summary of IC_50_ and Hill coefficient values obtained for *E. foeminea* extract treatments.

Treatment	Time (h)	IC_50_ ± Std.Error (*μ*g/mL)	Hill coefficient ± Std.Error	Sy.x∗
EtOAc	24	224.6 ± 1.234	3.143 ± 1.941	31.92
	48	161.6 ± 1.164	3.738 ± 1.639	23.80
	72	80.4 ± 1.221	1.401 ± 0.402	19.43

EtOH	24	98.45 ± 1.541	0.645 ± 0.275	24.5
	48	91.21 ± 1.220	1.082 ± 0.267	16.49
	72	167.4 ± 1.139	1.554 ± 0.307	13.61

H_2_O	24	78.01 ± 1.894	0.967 ± 0.723	47.7
	48	29.12 ± 1.434	0.751 ± 0.205	15.96
	72	28.23 ± 1.522	0.716 ± 0.217	18.08

Cisplatin	72	3.473 ± 1.106	2.108 ± 0.340	12.58

^∗^Standard deviation of the residuals in units of the *y*-axis of the dose-response curves.

**Table 2:** mRNA steady-state gene expression for markers involved in cell proliferation, apoptosis, and migration shown as fold change relative to that observed in untreated cells.

Gene	EtOH		H_2_O		EtOAc	
	Fold change	*P* value	Fold change	*P* value	Fold change	*P* value
Twist-1	-19.360	<0.001	-4.870	<0.001	-6.33	<0.001
p53	-16.038	<0.001	-4.166	<0.001	-1.857	0.035
MMP2	-14.657	<0.001	-4.383	<0.001	-1.994	0.131
MMP1	-7.177	<0.001	-3.658	<0.001	-1.181	0.220
BAX	-6.471	<0.001	-4.172	<0.001	-3.011	0.027
N-cadherin	-6.329	<0.001	-2.966	0.001	-1.181	0.220
B-catenin	-5.601	<0.001	-4.503	<0.001	-1.533	0.299
RUNX2	-5.211	<0.001	-2.993	0.001	-1.918	0.166
Vimentin	-4.189	<0.001	-3.265	0.001	—	—

**Table 3:** Comparison of U2OS gene expression when treated with either *E. foeminea*, doxorubicin, or nutlin-3.

Treatment	Downregulated genes		Upregulated genes	
*E. foeminea*∩doxorubicin∩nutlin-3	RUNX2	—	—	—

*E. foeminea*∩doxorubicin	B-catenin	N-cadherin	—	—

*E. foeminea*∩nutlin-3	Twist-1	—	—	—

*E. foeminea*	MMP2	BAX	—	—
	TP53	MMP1	—	—
	Vimentin	—	—	—

Doxorubicin∩nutlin-3	DCHS2	BIRC5	FAT3	BAX
	FAT4	CDH12	PIDD1	CDH10
	SOX2	CDH18	TP53I11	CDH22
	—	—	TP53I3	CDIP1
	—	—	TP53INP1	

Doxorubicin	XIAP	CBY1	LOC100653137///CDH23	CDH9
	CPED1	CDH4	MIR6513///TMBIM1	CDHR1
	CTNNA1	CELSR3	TMBIM6	CDHR3
	CTNND2	CPED1	TP53AIP1	CELSR1
	FAT1	TP53BP1	TP53I13	CELSR2
	FAT3	JMY	TP53INP2	CTNNB1
	TMBIM4	—	Twist-1	DACT1
	TMX2-CTNND1///CTNND1	—	TWIST2	DACT3

Nutlin-3	CELSR2	CDH24	CBY1	JMY
	DACT1	CDH6	CDH11	LINC-PINT
	DCHS1	CDHR2	CDH13	TWISTNB
	LOC100653137///CDH23	—	CDH15	XIAP
	—	—	CDH16	—

## Data Availability

The biological activity data used to support the findings of this study are included both within the article and in the supplementary material. All microarray datasets used in this study were obtained from the Gene Expression Omnibus (GEO) database, and all accession numbers are included in Table S2 in the supplementary material. Inquiries regarding raw data used in this study can be made through the corresponding author, Asma Amleh; aamleh@aucegypt.edu.

## Supplementary Materials

Supplementary Materials Figure S1. U2OS growth curve as determined over a 6-day period. The growth curve exhibited a sigmoid pattern with lag, exponential and statinary phases. Figure S2: Effect of E. foeminea ethyl acetate, ethanol and water extract treatments on U2OS cell morphology. Cells mostly possessed an elliptical shape in which they appeared wider at the center with tapering ends (red circles). Table S1. RT-PCR Forward (F) and Reverse (R) Primer Sequences, Annealing Temperature, Cycle Number and Amplicon Sizes.Table S2. Microarray Dataset Table S3. Summary of MTT assay Pvalues obtained for all extract treatments. Supplementary File 1.Fold change values of genes after doxorubicin treatment. Supplementary File 2.Fold change values of genes after nutlin treatment.
