# Supplementary material for: Potential Anticancer Activity of Crude Ethanol, Ethyl Acetate, and Water Extracts of Ephedra foeminea on Human Osteosarcoma U2OS Cell Viability and Migration
Source: Biomed Res Int. 2020 Jul 9;2020:3837693. doi: 10.1155/2020/3837693 (PMC7368211; doi:10.1155/2020/3837693)
Supplement: Supplementary Materials — Figure S1. U2OS growth curve as determined over a 6-day period. The growth curve exhibited a sigmoid pattern with lag, exponential and statinary phases. Figure S2: Effect of E. foeminea ethyl acetate, ethanol and water extract treatments on U2OS cell morphology. Cells mostly possessed an elliptical shape in which they appeared wider at the center with tapering ends (red circles). Table S1. RT-PCR Forward (F) and Reverse (R) Primer Sequences, Annealing Temperature, Cycle Number and Amplicon Sizes.Table S2. Microarray Dataset Table S3. Summary of MTT assay Pvalues obtained for all extract treatments. Supplementary File 1.Fold change values of genes after doxorubicin treatment. Supplementary File 2.Fold change values of genes after nutlin treatment. [file 3837693.f1.zip › Table S2..docx]

**Table S2.** Microarray Datasets

| Gene Expression Omnibus Sample | Description of U20S Cells Treatment |
| --- | --- |
| GSM1131226 | No treatment repeat 1 |
| GSM1131227 | No treatment repeat 2 |
| GSM1131228 | No treatment repeat 3 |
| GSM1131229 | DXR‑treated repeat 1 |
| GSM1131230 | DXR‑treated repeat 2 |
| GSM1131231 | DXR‑treated repeat 3 |
| GSM1131232 | DMSO‑treated repeat 1 |
| GSM1131233 | DMSO‑treated repeat 2 |
| GSM1131234 | DMSO‑treated repeat 3 |
| GSM1131235 | Nutlin‑3‑treated repeat 1 |
| GSM1131236 | Nutlin‑3‑treated repeat 2 |
| GSM1131237 | Nutlin 3 treated repeat 3 |

DXR (doxorubicin); DMSO (dimethyl sulfoxid)
